# Effects of Different Plyometric Training Frequencies on Components of Physical Fitness in Amateur Female Soccer Players

**DOI:** 10.3389/fphys.2018.00934

**Published:** 2018-07-17

**Authors:** Rodrigo Ramirez-Campillo, Felipe García-Pinillos, Amador García-Ramos, Javier Yanci, Paulo Gentil, Helmi Chaabene, Urs Granacher

**Affiliations:** ^1^Laboratory of Measurement and Assessment in Sport, Department of Physical Activity Sciences, Research Nucleus in Health, Physical Activity and Sport, Universidad de Los Lagos, Osorno, Chile; ^2^Department of Physical Education, Sports and Recreation, Universidad de La Frontera, Temuco, Chile; ^3^Department of Physical Education and Sport, Faculty of Sport Sciences, University of Granada, Granada, Spain; ^4^Department of Sports Sciences and Physical Conditioning, Faculty of Education, CIEDE, Catholic University of Most Holy Concepción, Concepción, Chile; ^5^Physical Education and Sport Department, Faculty of Education and Sport, University of the Basque Country (UPV/EHU), Vitoria-Gasteiz, Spain; ^6^Faculdade de Educacao Fisica e Danca, Universidade Federal de Goias, Goiania, Brazil; ^7^Division of Training and Movement Sciences, Research Focus Cognition Sciences, University of Potsdam, Potsdam, Germany; ^8^High Institute of Sports and Physical Education, Kef, University of Jendouba, Jendouba, Tunisia

**Keywords:** women, stretch-shortening cycle, muscle power, football, training load, agility

## Abstract

Plyometric jump training (PJT) is a frequently used and effective means to improve amateur and elite soccer players' physical fitness. However, it is unresolved how different PJT frequencies per week with equal overall training volume may affect training-induced adaptations. Therefore, the aim of this study was to compare the effects of an in-season 8 week PJT with one session vs. two sessions per week and equal training volume on components of physical fitness in amateur female soccer players. A single-blind randomized controlled trial was conducted. Participants (*N* = 23; age, 21.4 ± 3.2 years) were randomly assigned to a one session PJT per-week (PJT-1, *n* = 8), two sessions PJT per-week (PJT-2, *n* = 8) or an active control group (CON, *n* = 7). Before and after training, participants performed countermovement jumps (CMJ), drop-jumps from a 20-cm drop-height (DJ20), a maximal kicking velocity test (MKV), the 15-m linear sprint-time test, the *Meylan* test for the assessment of change of direction ability (CoDA), and the Yo-Yo intermittent recovery endurance test (Yo-YoIR1). Results revealed significant main effects of time for the CMJ, DJ20, MKV, 15-m sprint, CoDA, and the Yo-YoIR1 (all *p* < 0.001; *d* = 0.57–0.83). Significant group × time interactions were observed for the CMJ, DJ20, MKV, 15-m sprint, CoDA, and the Yo-YoIR1 (all *p* < 0.05; *d* = 0.36–0.51). *Post-hoc* analyses showed similar improvements for PJT-1 and PJT-2 groups in CMJ (Δ10.6%, *d* = 0.37; and Δ10.1%, *d* = 0.51, respectively), DJ20 (Δ12.9%, *d* = 0.47; and Δ13.1%, *d* = 0.54, respectively), MKV (Δ8.6%, *d* = 0.52; and Δ9.1%, *d* = 0.47, respectively), 15-m sprint (Δ8.3%, *d* = 2.25; and Δ9.5%, *d* = 2.67, respectively), CoDA (Δ7.5%, *d* = 1.68; and Δ7.4%, *d* = 1.16, respectively), and YoYoIR1 (Δ10.3%, *d* = 0.22; and Δ9.9%, *d* = 0.26, respectively). No significant pre-post changes were found for CON (all *p* > 0.05; Δ0.5–4.2%, *d* = 0.03–0.2). In conclusion, higher PJT exposure in terms of session frequency has no extra effects on female soccer players' physical fitness development when jump volume is equated during a short-term (i.e., 8 weeks) training program. From this, it follows that one PJT session per week combined with regular soccer-specific training appears to be sufficient to induce physical fitness improvements in amateur female soccer players.

## Introduction

Plyometric jump training (PJT) is widely and frequently used in soccer to improve players' physical fitness (Sedano Campo et al., 2009; Ozbar et al., 2014; Ramirez-Campillo et al., 2016c). In general, PJT involves quick and powerful multi-joint movements like jumping, hopping, and skipping. These movements are characterized by rapid eccentric phases, immediately followed by high-velocity concentric muscular actions that are potentiated through the stretch reflex (Taube et al., 2012). More specifically, there is ample evidence that PJT is beneficial to improve components of physical fitness in female soccer players (Sedano Campo et al., 2009; Ozbar et al., 2014; Ramirez-Campillo et al., 2016c; Ramrez-Campillo et al., 2016a). These benefits range from increases in lower muscle power, speed (i.e., linear sprint), change of direction ability (CoDA), kicking distance and velocity, repeated-sprint performance, aerobic endurance, and body composition (Sedano Campo et al., 2009; Ozbar et al., 2014; Ramirez-Campillo et al., 2016c; Ramrez-Campillo et al., 2016a). Of note, these adaptive processes constitute key physical fitness characteristics that, in addition to technical and tactical qualities, contribute to performance in female soccer (Arnason et al., 2004; Spencer et al., 2005; Datson et al., 2014). PJT-related physiological adaptations primarily involve the central and peripheral nervous systems (Markovic and Mikulic, 2010; Taube et al., 2012). In addition, there is evidence for somatic and musculoskeletal adaptations (Sedano Campo et al., 2009; Markovic and Mikulic, 2010; Alvarez et al., 2012). However, the optimal PJT design that improves components of physical fitness in amateur female soccer still needs to be elucidated (Ramirez-Campillo et al., 2018a). In a recent scoping review (Ramirez-Campillo et al., 2018a) identified four randomized controlled trials only that examined the effects of PJT in female soccer players. These studies adopted different methodological training approaches (Sedano Campo et al., 2009; Ozbar et al., 2014; Ramirez-Campillo et al., 2016c; Ramrez-Campillo et al., 2016a), which do not allow to elucidate optimal PJT program parameters for female soccer players. Thus, further comparative studies are needed to elucidate optimal PJT modalities (i.e., intensity, volume, frequency, etc.) for this group of athletes.

An important PJT modality is training frequency (Ramirez-Campillo et al., 2018a), usually reported as the number of training sessions performed during a given period of time (i.e., 1 week). A previous study (Sedano Campo et al., 2009) examined the effects of a 12 week PJT with 3 sessions per week in amateur female soccer players aged 23 years and observed significant improvements in countermovement jump (CMJ; Δ14.5%), drop-jump (DJ; Δ16.1%), and maximal kicking velocity (MKV) performance in the dominant (Δ11.9%) and non-dominant leg (Δ13.2%). In another study (Ozbar et al., 2014), 8 weeks of PJT with a frequency of one session per week significantly improved triple-jump distance of the dominant (Δ12.1%) and non-dominant leg (Δ15.7%), standing broad-jump distance (Δ5.2%), CMJ height (Δ17.6%), CMJ peak power (Δ10.8%), and 20-m sprint-time (Δ8.1%) in amateur female soccer players aged 18 years. Following 6 weeks of PJT with two sessions per week, a previous work (Ramirez-Campillo et al., 2016c) demonstrated significant improvements in CMJ height (Δ10.7%), CMJ height with arm swing (Δ8.3%), the reactive-strength index (Δ21.5%), the medicine ball throwing test (Δ6.7%), the 30-m sprint-time test (Δ5.2%), a CoDA test (Δ4.0%), and aerobic performance in the 20-m shuttle run test (Δ9.7%) in amateur female soccer players aged 22 years.

Of note, the effects of different PJT frequencies have previously been analyzed in futsal players (Yanci et al., 2017), and in prepuberal male soccer players (Bouguezzi et al., 2018). Yanci et al. (2017) examined the effects of one vs. two PJT sessions on components of physical fitness in male futsal soccer players aged 24 years while controlling for weekly training volume. It was found that two PJT sessions per week significantly improved CoDA performance (Δ4.8%) and vertical as well as horizontal jump performance (Δ3.4 to Δ15.3%). One PJT session per week improved 15-m linear sprint-time (Δ2.4%) and repeated-sprint performance (Δ2.4 to Δ5.1%). Bouguezzi et al. (2018) studied the effects of an 8-week PJT with either one vs. two sessions per week on components of physical fitness in prepuberal soccer players. These authors reported comparable performance improvements irrespective of training frequency in measures of muscle strength and power, CoDA performance, and kicking distance. However, none of the aforementioned studies examined the effects of different PJT frequencies on components of physical fitness in adult female soccer players. Considering that the effects of PJT could partially be moderated by factors such as sex (De Villarreal et al., 2009) and age (i.e., maturation) (Asadi et al., 2017, 2018; Moran et al., 2017,a,b).. , further research is needed to elucidate the effects of different PJT frequencies in adult female athletes.

It has previously been reported for resistance training (Dankel et al., 2017) that higher vs. lower training frequencies may produce greater training-induced adaptations. Considering the lack of data on the effects of different PJT training frequencies on components of physical fitness in female soccer players, we aimed at examining the effects of 8 weeks PJT with either one or two sessions per week and equal training volume on components of physical fitness (i.e., muscle strength and power, speed, CoDA, aerobic endurance) in amateur female soccer players. With reference to previous findings (De Villarreal et al., 2009; Saez De Villarreal et al., 2012; Dankel et al., 2017; Yanci et al., 2017), we hypothesized that two PJT sessions per week would be more effective than one session per week to improve physical fitness in amateur female soccer players.

## Materials and methods

A single-blind randomized controlled trial was conducted to compare the effects of an in-season 8 weeks PJT with one vs. two sessions per week and equal training volume on components of physical fitness in amateur female soccer players. Pre- and post-performance assessment consisted of the CMJ test, the drop-jump test from a 20-cm drop-height (DJ20), the maximal kicking velocity (MKV) test, the 15-m linear sprint-time test, the *Meylan* CoDA test, and the Yo-Yo intermittent recovery endurance test level 1 [Yo-YoIR1]). Two weeks prior to the start of the study, we conducted two familiarization sessions with 30-min each to explain and perform test and training procedures to all participants in order to reduce potential learning effects.

### Participants

Twenty-three amateur female soccer players aged 21.4 ± 3.2 years were recruited from a regional-level soccer team. They were randomly assigned to a PJT with one session per week (PJT-1, *n* = 8), two sessions per week (PJT-2, *n* = 8) or an active control group (CON, *n* = 7). The randomization sequence was generated electronically (https://www.randomizer.org) and was concealed until interventions were assigned. Participant characteristics are provided in Table 1. The sample size was determined using the following online analysis tool: http://www.statisticalsolutions.net/pssZtest_calc.php. The a priori power analysis revealed that a total of 7 participants per-group is needed to yield a power of 80% at the α = 0.05 level.

**Table 1:** Descriptive data of the control group, PJT-1, and PJT-2 groups.

	**Control** **(*n* = 7)**	**PJT-1** **(*n* = 8)**	**PJT-2** **(*n* = 8)**
Age (y)	20.1, 1.8	22.8, 4.3	21.4, 2.5
Body mass (kg)	55.3, 3.3	54.9, 3.7	59.6, 8.5
Height (cm)	160.1, 5.0	158.0, 3.0	157.6, 4.8
Body mass index (kg.m^−2^)	21.6, 1.4	22.0, 1.6	24.0, 3.2
Body fat (%)	26.6, 2.2	24.0, 2.5	25.1, 6.7
Body water (%)	49.1, 2.2	51.2, 4.2	49.6, 7.1
Muscle mass (%)	35.4, 1.9	35.9, 0.9	34.5, 3.4
Bone mass (%)	10.1, 0.6	10.9, 1.0	10.5, 0.8
Soccer training load (AU)^a^	309, 117	510, 332	420, 287
Years of soccer experience (y)	6.0, 1.6	5.4, 1.4	5.6, 1.8

*PJT-1 and PJT-2: plyometric jump training groups using a training frequency of one or two weekly sessions, respectively*.

a *Soccer training load (in arbitrary units, AU) was determined by multiplying the minutes of soccer training by the rating of perceived exertion after two randomly selected training sessions (soccer matches not-included)*.

Upon recruitment, players completed three 120-min soccer-training sessions and a competitive match per week. Players had similar competitive schedules and comparable soccer drill programs, resulting in similar soccer-specific weekly training loads (Table 1). The training groups conducted a PJT program in replacement for some technical-tactical soccer drills, whereas the CON followed their regular soccer-specific training program. All participants met the following inclusion criteria: (i) a background of ≥2 years of systematic soccer training and competitive experience, (ii) continuous soccer training with no musculoskeletal injuries during the last 2 months prior to the start of the study, (iii) no systematic PJT experience during the last 5 months, (iv) absence of potential medical problems that could compromise participation or performance in the study, and (v) absence of any lower-extremity surgery in the two last years. Similar numbers of goalkeepers (1; 0; 1), defenders (2; 3; 2), midfielders (3; 2; 2), and forwards (2; 3; 2) were present in the PJT-1, PJT-2, and CON, respectively.

This study was carried out in accordance with the recommendations of the latest Declaration of Helsinki. The protocol was approved by the review board from the Department of Physical Activity Sciences, University of Los Lagos. Participants were informed about the experimental procedures and its possible harms and benefits before the start of the study. Written informed consent was obtained from all participants before the beginning of the study.

### Experimental design

Before and after training, physical fitness tests were scheduled ≥72 h after a soccer match or training session. Tests were always completed on the same grass soccer field between 3 and 6 p.m. where players trained and competed. During pre- and post-tests, the wind velocity ranged between 9.1 and 11.9 km.h^−1^, the relative humidity amounted to 55–64%, and the temperature was between 12 and 17°C (local Meteorological Service). Participants used the same soccer sports clothes that they usually wear during training and competitions. The same investigator, who was blinded to group allocation, conducted all measurements. Participants were asked to perform at their maximum effort during testing. Players were evaluated over 2 days. On the first day, data on age, stature, body mass and composition, and total years of soccer training were recorded. During the intervention period, neither of the participants were involved in the practice of other sports nor in soccer training with other clubs. During the first day, participants performed the CMJ test, DJ20 test, and the MKV test. On the second day, they performed the 15-m linear sprint test, the *Meylan* CoDA test, and the Yo-YoIR1 test. The highest score from three trials was recorded for all tests except the Yo-YoIR1 test. For the later, only one all-out single trial was performed (Hopkins, 2000; Ramirez-Campillo et al., 2016c). A rest interval of at least 2-min was provided between each physical fitness trial. While waiting, participants performed low-intensity activities (i.e., walking, ball passing) to maintain their readiness for the next test. Ten minutes of general (i.e., submaximal running with changes of direction) and specific (Andrade et al., 2015) (vertical and horizontal submaximal jumps) exercises were performed before each test session as a warm-up. In addition, participants performed a test-specific warm-up that comprised two practice jumps, runs, or kicks.

### Anthropometry

Stature was measured using a stadiometer (Bodymeter 206, SECA®, Hamburg, Germany to 0.1 cm) and body mass and composition on an electrical scale (InBody120, model BPM040S12FXX, Biospace, Inc., Seoul, Korea, to 0.1 kg).

### Vertical jump tests

All jump tests were performed according to previous recommendations (Ramirez-Campillo et al., 2013, 2015b,d). Briefly, for the vertical jumps (i.e., CMJ, DJ20), players executed maximal-effort jumps on a validated mobile contact mat (Ergojump® Globus, Codogne, Italy) with arms akimbo (Pueo et al., 2017). Take-off and landing were standardized to the same spot and players were required to perform full knee and ankle extensions during the flight phase. Participants were instructed to maximize their jump height. For the DJ20 test, players were instructed to minimize ground contact time (<250-ms) after dropping down from a 20-cm box.

### Maximal kicking velocity

The MKV test was performed as previously outlined (Ramirez-Campillo et al., 2015b). Participants performed a maximal instep kick with their dominant leg after a run-up of two strides, using a size five soccer ball (Adidas Starlancer V®, FIFA certified). Maximal velocity was measured using a radar gun (Speed Gun SR3600; Sports Radar®, Homosassa, Florida, USA).

### Linear sprint and *meylan* CoDA performances

Sprint time was assessed to the nearest 0.01 s using single-beam timing gates (Brower® Timing System, Salt Lake City, Utha, USA). Participants started in standing position with the toe of the preferred foot placed behind the starting line. The sprint started when the athlete voluntarily initiated the test, which triggered timing automatically. The timing gates were positioned at the beginning (0.3 m in front of the athlete) and at 15 m and were set ~0.7 m above the floor (i.e., hip level). This system enables capturing trunk movement rather than a false trigger from a limb. For the *Meylan* CoDA test, the timing system and procedures were the same as for the 15-m linear sprint test except that players had to run as quickly as possible while performing several changes of direction (Meylan and Malatesta, 2009).

### Yo-Yo intermittent recovery level-1 endurance test (Yo-YoIR1)

The test was executed as previously described (Krustrup et al., 2003). Before testing, participants performed a warm-up consisting of the first 4 running bouts in the test.

### Monitoring of training load

Soccer training load was measured during two randomly assigned training sessions. Session rating of perceived exertion (RPE) was measured as previously described (Ramirez-Campillo et al., 2014a). Briefly, each player's RPE was collected ~30-min after each soccer training session to ensure that the perceived effort reflected the entire session rather than the most recent exercise intensity. Total training load was calculated as the RPE × training session duration (i.e., minutes). Athletes regularly used the RPE scale during training so that they were accustomed to it.

### Training program

The PJT program was completed during the mid-portion of the competitive season. The drills, sets, repetitions, and progressions per week are detailed in Table 2. The active CON followed their regular soccer training (i.e., mainly technical-tactical drills, small-sided and simulated games, and injury prevention drills). The design of the PJT intervention was based on the players' previous training records and research results (Ramirez-Campillo et al., 2015a, 2018a) in order to include unilateral, bilateral, cyclical (i.e., repeated), acyclical (i.e., non-repeated), vertical, horizontal, and turn jumps, in addition to fast (< 250-ms of foot-ground contact-time) and slow (≥250-ms of foot-ground contact-time) stretch-shortening cycle (SSC) muscle contractions, with a strong emphasis on landing technique and shock absorption, using both soft and medium-hard training surfaces (Ramirez-Campillo et al., 2013; Kibele et al., 2014; Granacher et al., 2015). The PJT was not added to the regular soccer training but was immediately performed after the warm-up program (Ramirez-Campillo et al., 2018c) in replacement of some low-intensity technical-tactical soccer drills. In this context, the PJT was performed within the regular 120-min soccer training period, once (PJT-1) or twice (PJT-2) per week during the 8 weeks intervention period. The PJT replacement activity accounted for < 6% of the total soccer-training load (competitive and friendly matches not accounted for). Each PJT session included one set of 5 different jump drills, with 7–14 repetitions per-set for the PJT-2 group and 14–28 repetitions per set for the PJT-1 group. The jump drills included DJs, standing long-jumps (SLJ), unilateral CMJs, 180° jumps, and repeated CMJs. The DJs and the repeated CMJs were performed with arms akimbo to facilitate fast-SSC, and the other jump drills were performed using arm swing. During vertical and horizontal jumping, participants were encouraged to achieve maximal vertical height and horizontal distance, respectively. During DJs, participants were instructed to maximize the ratio between vertical height and ground contact-time. It is noteworthy that participants used individualized box heights (i.e., 5–35 cm) during DJs as previously suggested (Ramirez-Campillo et al., 2018b).

**Table 2:** Plyometric jump training program^¥^.

	**Weeks 1–2**	**Weeks 3–4**	**Weeks 5–6**	**Week 7**	**Week 8**
Drop jump^*^ (repetitions)	8–16^*^	10–20	12–24	14–28	7–14
Standing long jump (repetitions)	8–16	10–20	12–24	14–28	7–14
Unilateral countermovement jump (repetitions)	8–16	10–20	12–24	14–28	7–14
180° jump (repetitions)	8–16	10–20	12–24	14–28	7–14
Repeated countermovement jump (repetitions)	8–16	10–20	12–24	14–28	7–14

¥ The order of execution for the jump drills was randomized each week to provide variation to training;

* *Repetition values represent the number of jumps per training session. Values at the left and the right correspond to the plyometric jump training groups that used two or one training sessions per week*.

For the DJs, the technique of execution was the same as the one described previously (i.e., section Vertical Jump Tests). For the SLJ, participants positioning their feet shoulder-width apart, and performed a fast downward movement (~120° knee angle), followed by a maximal-effort horizontal jump. During the unilateral CMJ, the participants started with the foot of the designated leg on the ground, then performed a moderate-speed downward movement, and then jumped as high as possible with arm swing. In the 180° jump, participants started in a standing erect position with feet shoulder-width apart, and then performed a 2-footed vertical jump, combined with a 180° rotation in midair, keeping the arms away from her sides to help maintain balance, and striving to achieve maximal height. At landing, participants reverses this jump into the opposite direction. For the repeated CMJs, participants performed the initial jump with the intention to reach maximal vertical jump height, and the remaining jumps with the intention to maximize reactive strength.

The reliability of jump heights and jump contact-times for selected drills was verified in a randomly assigned subsample of participants (two from each group) during two randomly assigned training sessions using the same procedure as described above. Briefly, the maximal intensity for CMJ and DJ20 was verified by measuring either height or ground contact-time of the respective drill with an electronic contact mat (Ergojump; Globus, Codogne, Italy).

The order of drills was randomized during each week to provide variation to training and to avoid monotony. As previously suggested (Ramirez-Campillo et al., 2015c), the volume of training was increased progressively from the 1st week, toward the 7th week of training. During the 8th week, a taper strategy in terms of training volume was applied. An investigator to participant ratio of 1:5 was realized during all training sessions and particular attention was paid to the technical execution of jumps. All plyometric sessions lasted ~6–20-min. The training sessions for PJT-1 were two times longer than the sessions for PJT-2 because of the greater training volume per session. However, the total number of jumps performed during the whole training intervention (i.e., 8 weeks) was similar for both groups. This is because the number of jumps performed during one PJT-1 session was equally distributed into two sessions for PJT-2. That is, both groups performed a total of 810-foot contacts per leg after completion of the program, with the same weekly progressive overload and taper strategies. In addition, both PJT groups trained at the same time of day (6–8 p.m.), with the same rest intervals between drills sets (i.e., 30–60 s) (Ramirez-Campillo et al., 2014a), and jumps (i.e., 5–15 s for acyclic jumps) (Ramirez-Campillo et al., 2014b). The only between-group difference was that PJT-1 conducted one PJT session per-week while PJT-2 performed two PJT sessions per week. During each session, different training surfaces (i.e., grass, land-dirt) were used. All training surfaces were available in the same outdoor soccer field. The order of the applied surface was randomized each week with an equal surface distribution.

### Statistical analysis

Data are presented as group mean values and standard deviations. After data normality assumption was verified with the Shapiro-Wilk test, analyses of variance (ANOVA) were used to detect baseline between group differences and training effects over time. Dependent variables were analyzed in separate 3 (Groups: PJT-1, PJT-2, CON) × 2 (Time: pre, post) ANOVA with repeated measures on time. *Post-hoc* tests with Bonferroni-adjusted α were conducted to identify comparisons that were statistically significant. Effect sizes were determined by calculating Cohen's *d-*values (Cohen, 1988). A detectable effect size (*d*) of 0.2 (Hopkins, 2004), as previously suggested after the application of comparable PJT programs (Radnor et al., 2017), was considered as meaningful (i.e., smallest worthwhile change). Statistical analyses were carried out using STATISTICA statistical package (Version 8.0; StatSoft, Inc., Tulsa, USA). Significance levels were set at α = 5%. To assess test-retest reliability, thresholds for the intra-class correlation coefficient were set at ≥0.80 (Hopkins, 2000).

## Results

Test-retest reliability was above the established threshold and ranged from 0.81 to 0.95 according to the intra-class correlation coefficient. Moreover, participants achieved a mean of 196 beats.minute^−1^ at the end of the single-trial maximal effort Yo-YoIR1 test.

Initially, 29 participants fulfilled the inclusion criteria and were selected to participate in this study. To be included in the final analyses, each participant needed to complete ≥87% of all training sessions (i.e., 14 out of 16 sessions for PJT-2 and 7 out of 8 sessions for PJT-1) and attend pre and post tests. Because of these strict requirements, 6 participants were excluded from our final data analysis.

All participants received treatments as allocated. Between-group soccer-specific training adherence rates were similar (CON: 87.6 ± 6.1%; PJT-1: 84.5 ± 7.8%; and PJT-2: 88.0 ± 9.2%). Additionally, PJT adherence rate was the same for PJT-1 and PJT-2 (93.7 ± 5.8%). Moreover, although the training load monitoring did not consider the load of competitive matches, players from the PJT-1, PJT-2, and active CON achieved similar competitive loads in terms of number of competitive matches played (matches played = 7.5 ± 0.8, 7.0 ± 1.7, and 7.5 ± 0.5, respectively). In addition, the soccer training load (Table 1) was not significantly different (*p* = 0.2 to *p* = 0.5) between the groups.

No significant baseline between-group differences were recorded for all somatic and fitness measurements (all *p* > 0.05, *d* = 0.02–0.23) (Table 3). The main effects of group, time, and the group × time interactions are displayed in Table 3.

**Table 3:** Group specific means and standard deviations (SD) of physical fitness outcome measures before (Pre) and after (Post) training.

	**Control (*****n*** = **7)**		**PJT-1 (*****n*** = **8)**		**PJT-2 (*****n*** = **8)**		**ANOVA outcomes**		
	**Pre**	**Post**	**Pre**	**Post**	**Pre**	**Post**	**Group *F*_(2, 20)_, *p*-value (*d*)**	**Time *F*_(1, 20)_, *p*-value (*d*)**	**Group × Time *F*_(2, 20)_, *p*-value (*d*)**
Countermovement jump (cm)	28.8 ± 4.9	29.9 ± 5.1	28.5 ± 6.9	31.5 ± 7.5^¥^	27.4 ± 4.3	30.1 ± 4.7^¥^	*F* = 0.1, *p* = 0.91 (0.01)	*F* = 95.3, *p* < 0.001 (0.83)	*F* = 5.7, *p* < 0.02 (0.36)
20-cm drop jump (cm)	28.7 ± 4.3	29.3 ± 4.6	27.2 ± 5.9	30.9 ± 7.8^¥^	27.7 ± 5.8	31.3 ± 6.6^¥^	*F* = 0.1, *p* = 0.98 (0.01)	*F* = 76.8, *p* < 0.001 (0.79)	*F* = 10.2, *p* < 0.001 (0.51)
Maximal kicking velocity (km.h^−1^)	67.3 ± 7.2	68.9 ± 7.5	65.1 ± 9.0	70.6 ± 8.9^¥^	63.0 ± 9.5	68.9 ± 11.0^¥^	*F* = 0.1, *p* = 0.88 (0.01)	*F* = 88.3, *p* < 0.001 (0.82)	*F* = 8.6, *p* < 0.01 (0.46)
15-m sprint time (s)	3.42 ± 0.2	3.45 ± 0.2	3.28 ± 0.1	3.01 ± 0.1^¥^	3.43 ± 0.1	3.10 ± 0.1^¥^	*F* = 11.9, *p* < 0.01 (0.54)	*F* = 26.7, *p* < 0.001 (0.57)	*F* = 8.9, *p* < 0.01 (0.47)
Change of direction speed time test (s)	4.96 ± 0.2	4.95 ± 0.4	4.94 ± 0.2	4.57 ± 0.2^¥^	5.12 ± 0.3	4.74 ± 0.3^¥^	*F* = 1.5, *p* = 0.26 (0.13)	*F* = 29.5, *p* < 0.001 (0.61)	*F* = 6.2, *p* < 0.01 (0.38)
Yo-Yo intermittent recovery test (m)	606 ± 175	612 ± 179	573 ± 237	628 ± 244^¥^	630 ± 192	690 ± 203^¥^	*F* = 0.2, *p* = 0.83 (0.02)	*F* = 53.1, *p* < 0.001 (0.73)	*F* = 9.4, *p* < 0.01 (0.49)

*PJT-1 and PJT-2: plyometric jump training groups using a training frequency of one or two weekly training sessions; d: Cohen's d*;

¥ *Significant pre-post change (p < 0.01)*.

### Effects on components of physical fitness

Statistical results revealed significant main effects of time (all *p* < 0.001; *d* = 0.57–0.83) and group × time interactions (all *p* < 0.05; *d* = 0.36–0.51) for the CMJ, DJ20, MKV, 15-m linear sprint, *Meylan* CoDA, and Yo-YoIR1 tests (Table 3). For PJT-1 and PJT-2, *post-hoc* analyses revealed similar significant improvements in CMJ (Δ10.6%, *d* = 0.37; and Δ10.1%, *d* = 0.51, respectively), DJ20 (Δ12.9%, *d* = 0.47; and Δ13.1%, *d* = 0.54, respectively), MKV (Δ8.6%, *d* = 0.52; and Δ9.1%, *d* = 0.47, respectively), 15-m linear sprint (Δ8.3%, *d* = 2.25; and Δ9.5%, *d* = 2.67, respectively), *Meylan* CoDA (Δ7.5%, *d* = 1.68; and Δ7.4%, *d* = 1.16, respectively), and Yo-YoIR1 (Δ10.3%, *d* = 0.22; and Δ9.9%, *d* = 0.26, respectively) performances. No significant pre-post changes were observed for CON (all *p* > 0.05; Δ0.5–4%, *d* = 0.03–0.2).

## Discussion

The aim of the present study was to compare the effects of an 8 week in-season PJT with one session vs. two sessions per week and equal weekly training volume on measures of physical fitness (i.e., CMJ, DJ20, MKV, 15 m sprint, CoDA, and Yo-YoIR1 tests) in amateur female soccer players. Our main findings showed that both training interventions were equally effective to improve key components of physical fitness in amateur female soccer players when controlled for training volume. Therefore, a higher weekly in-season PJT training frequency during 8 weeks has no extra effects on female soccer players' physical fitness development.

Results demonstrated that both PJT groups improved vertical (i.e., CMJ and DJ20) jump performance (Table 3). These findings corroborate previous research that reported improvements (*d* = 0.23–0.61) in vertical jump performances in female soccer players after PJT (Ramirez-Campillo et al., 2016c; Ramrez-Campillo et al., 2016a). In addition, the present findings confirm results from previous reviews and meta-analyses that indicated the beneficial effects of PJT on vertical jump performance in soccer players (García-Ramos et al., 2017). However, this is the first study that compared the effects of an 8 week PJT with either one or two weekly training sessions on vertical jump performance of senior female amateur soccer players. Considering that, aside from technical-tactical qualities, jumping performance is a key proxy of competitive soccer athletic performance (Arnason et al., 2004), the present results have important practical implications. The distribution of training volume on either one or two weekly training sessions yielded similar effects on vertical jump performance for slow-SSC jumps (i.e., CMJ) and fast-SSC jumps (i.e., DJ20). The observed performance enhancements could be caused by various neuromuscular adaptations, such as inter-muscular coordination improvements, increased alpha motor-neurons firing rate, improved mechanical characteristics of the muscle-tendon complex, improved muscle size, architecture and/or single-fiber mechanics (Markovic and Mikulic, 2010).

Concerning kicking velocity, 8 weeks of PJT in combination with soccer-specific training resulted in improvements in this physical fitness trait (PJT-1: *d* = 0.52; PJT-2: *d* = 0.47). This is in agreement with previous findings (Sedano Campo et al., 2009; Rubley et al., 2011). The PJT-related neuromuscular adaptations in terms of strength and power gains could be responsible for the observed improvements in the kicking velocity test (Lees et al., 2010; Markovic and Mikulic, 2010; Michailidis et al., 2013). To the authors' knowledge, only one study has previously analyzed the effects of PJT frequency on kicking performance (Bouguezzi et al., 2018). In the aforementioned study, an improvement (*d* = 1.83) was observed after 8 weeks of PJT with a training frequency of either one or two weekly training sessions. Compared to our study, the greater magnitude in performance improvement in the kicking velocity test reported by a previous work (Bouguezzi et al., 2018) may be explained by the younger age (maturity) of the examined soccer players (age, ~11.8 years; maturity-offset, −3.0 years). Thus, at a better receptive age for soccer-specific skills (Ford et al., 2011), the athletes in the study of Bouguezzi et al. (2018) may had greater potential to improve soccer-specific technical performance such as kicking the ball. However, a previous study with young soccer players (Ramirez-Campillo et al., 2015b) found improvements in MKV of similar magnitude (ES = 0.36–0.67) as those registered in the current study. An alternative explanation for the apparent discrepancy between our results and those from the aforementioned study (Bouguezzi et al., 2018), may be related to the test used to assess kicking performance. In our study we test for maximal kicking velocity, while in the aforementioned study (Bouguezzi et al., 2018) the tested for maximal kicking distance. In previous PJT studies with female soccer players (Sedano Campo et al., 2009; Rubley et al., 2011), athletes improved their kicking distance performance up to Δ22% (Rubley et al., 2011), whereas the improvement in the kicking velocity test, was only ~Δ12% (Sedano Campo et al., 2009). This seems to be due to the fact that kicking distance is influenced by numerous “external” factors such as the trajectory and rotation of the ball as well as the techique used to kick the ball (i.e., toe, dorsum or the inside part of the foot). This can be learned by the player and might influence kicking performance regardless of muscle power level.

The beneficial effect of in-season PJT on sprint-time performance in female soccer players has been well established (Δ3.2–8.1%; *d* = 0.35–0.86) (Ozbar et al., 2014; Ramirez-Campillo et al., 2016c; Ramrez-Campillo et al., 2016a). In view of the high frequency of short and high-intensity sprints during soccer matches, improving the quality of acceleration is paramount to increase soccer's chances of winning challenges (i.e., winning ball possession, stand out from opponents) in a real game situation (Vigne et al., 2010; Datson et al., 2014). Findings of the present study demonstrated that both interventions were effective in improving acceleration (i.e., 15-m sprint-time) performance after 8 weeks of training (Table 3). It must be noted that a meaningful portion of the PJT drills (3 out of 5; i.e., standing long jumps; unilateral CMJ; 180° jumps) in the current intervention implicated slow-SSC muscle actions. These actions mimic those encountered during the acceleration phase of a sprint (Mero, 1988; Mero et al., 1992) compared to the faster-SSC muscle actions of the maximal velocity phase of a sprint (Mero et al., 1992). In this sense, the specificity principle of training may help to explain the improvement in the 15 m sprint test after the PJT program (Rimmer and Sleivert, 2000). That is, greater improvements may occur when the testing procedure (i.e., 15 m sprint acceleration, involving relatively slow-SSC muscle actions) mimic the velocity of muscle action used during training (Behm and Sale, 1993), which in the current study involved mainly slow-SSC muscle actions. The underlying mechanisms of this specificity phenomenon may be of both neural and muscular nature (Behm and Sale, 1993; Markovic and Mikulic, 2010). Aside from the potential role of velocity-specificity principle of training to explain the improvement observed in 15 m sprint performance, the direction of application of muscle force may also help to explain the magnitude of current findings (Ramirez-Campillo et al., 2015b). In this sense, the inclusion of horizontal jump drills may have contributed to the 15 m sprint improvements, considering the importance of horizontal force production in sprint performance (Morin et al., 2012; Kawamori et al., 2013). It is worth noting that there are no studies dealing with the effect of manipulating PJT session frequency in female soccer players equating for total volume. A recent study (Yanci et al., 2017) compared two PJT frequencies (i.e., one vs. two sessions per week) equated for total volume during 6 weeks in adult male futsal soccer players and demonstrated significant performance improvement in CoDA, SLJ, and CMJ irrespective of PJT training group frequency. However, only players that trained once per week significantly improved their pre-post 15 m sprinting speed performance. In a recent study (Bouguezzi et al., 2018), prepubertal male soccer players completed an 8 week PJT program either with one or two training sessions per week equated for total volume, achieving similar improvements in 5 m sprint-time (*d* = 0.53). Considering the results from both previous studies and the current intervention, it seems that one or two PJT sessions per week induce similar improvements in acceleration sprint in female soccer players. This is consistent with the result obtained in female soccer players after PJT, where significant improvements were observed in sprint performance after interventions that used either one (Ozbar et al., 2014) or two training sessions per week (Ramirez-Campillo et al., 2016c; Ramrez-Campillo et al., 2016a).

The CoDA is a key proxy for soccer athletic performance (Reilly, 1996; Reilly et al., 2000; Datson et al., 2014). Improvements in CoDA in soccer players after PJT have been previously reported (Bedoya et al., 2015; Asadi et al., 2016). However, as a novelty, current findings indicate that 8 weeks of PJT either with one or two weekly training sessions induce similar CoDA improvements in adult female amateur soccer players (Table 3). In a previous study with male futsal players (Yanci et al., 2017), the distribution of PJT volume over 2 weekly training sessions induced significant improvements in CoDA (i.e., 505 test), whereas no significant improvements were observed when the same volume was distributed in only one weekly training session. However, the authors included specific CoDA drills during the intervention, which may have partially affected the study outcomes due to learning. In fact, in a study with young male soccer players (Bouguezzi et al., 2018), when the total volume of PJT was divided into one or two weekly training sessions during 8 weeks, without the incorporation of specific CoDA training drills, similar improvements were observed in CoDA. Considering the relevance of both vertical and horizontal force neuromuscular-generating capabilities (Young, 2006; Young and Farrow, 2006; Young et al., 2015), and the relevance of unilateral performance during CoDA movements (Young et al., 2002; Ramirez-Campillo et al., 2015a), especially in soccer (Datson et al., 2014; Meylan et al., 2014), the incorporation of vertical, horizontal, and unilateral drills in the current intervention probably increased the chances for improvements in CoDA. On the whole, it seems that changing PJT frequency (i.e., the number of training session per-week) does not yield any additional CoDA performance improvement in female soccer players when a moderate volume (i.e., 810 jumps per leg over 8 weeks) is applied. Therefore, different variables than PJT frequency (i.e., volume) may have a greater role in enhancing CoDA in female soccer players. PJT induced neuromuscular adaptations such as increased firing frequencies (Hakkinen et al., 1985) and increased reactive strength (Young et al., 2002) may help to explain the improvement in CoDA.

Our results demonstrated a significant (*d* = 0.22–0.26) increase in Yo-YoIR1 performance in both PJT groups (Table 3). Moreover, the relative (Δ) improvements in both the PJT-1 (10.3%) and the PJT-2 (9.9%) groups were ten-fold greater compared to the control group (1%). Further, when individual values were analyzed, in the control group only 3 participants improved Yo-Yo performance, 2 participants were able to maintain performance, and 2 participants actually reduced performance. On the contrary, all participants from the PJT groups improved Yo-Yo performance. Plyometric training may not induce a significant increase in underlying aerobic qualities such as maximal oxygen consumption (VO2max) (Barnes and Kilding, 2015; Blagrove et al., 2017) or lactate threshold (Gorostiaga et al., 2004; Blagrove et al., 2017), but has been shown to improve anaerobic performance (Assuncao et al, 2017) and may still have an effect on female soccer players on endurance performance tests with repeated changes of direction with intermittent recovery (Ramirez-Campillo et al., 2016c; Ramrez-Campillo et al., 2016a; Rosas et al., 2017). Of note, this seems to be the first study to report the positive effects of PJT on endurance performance with repeated changes of direction and with intermittent recovery, which might be more specific for soccer players (Krustrup et al., 2003). Moreover, this seems to be the first PJT study to report similar Yo-YoIR1 performance improvements after an equated volume of training distributed over one or two weekly training sessions. Improvements in explosive performance after PJT can contribute to the change of direction during an intermittent test (i.e., Yo-YoIR1) with change of direction (Buchheit, 2008) or to running economy (Blagrove et al., 2017), independently from the influence on VO2max (Balsalobre-Fernandez et al., 2016) or lactate metabolism (Spurrs et al., 2003). The improvement in DJ20 observed in the current study, reflecting an improved performance to produce maximal strength in a minimal time (Flanagan and Comyns, 2008) as a consequence of improved rate-of-force development and motor unit recruitment level (Markovic and Mikulic, 2010), may have transferred into improved running economy and enhance aerobic performance independently of others aerobic indicators (Coyle, 1995), especially considering the relevance of neuromuscular-mediated changes in the athletes' running economy (Yamamoto et al., 2008) or the neuro-mechanical improvements (Markovic and Mikulic, 2010) that may positively affect the athletes' change of direction endurance results (Buchheit, 2008). An increased tendon stiffness after PJT (Markovic and Mikulic, 2010), may also allow for a faster transfer of force from contracting muscles to moving bones through tendons (Legerlotz et al., 2016), positively affecting athletes' performance to change direction during an intermittent endurance test (Buchheit, 2008). Direct assessment of potential mechanisms that could improve endurance performance after PJT in female soccer players deserves further consideration by well-controlled studies.

Of note, although PJT can induce a broad range of adaptations (Markovic and Mikulic, 2010), several specific PJT characteristics should be considered in order to maximize its benefits (King and Cipriani, 2010). In this study, the PJT program included unilateral (Ramirez-Campillo et al., 2015a), bilateral (Ramirez-Campillo et al., 2015a), cyclical (Makaruk et al., 2014), acyclical (Komi, 2000), vertical (Ramirez-Campillo et al., 2015b), horizontal (Ramirez-Campillo et al., 2015b), and turn jumps (Hewett et al., 2005), in addition to fast and slow SSC muscle contractions (Bobbert, 1990), with a strong emphasis on landing technique and shock absorption, using both soft and medium-hard training surfaces (Ramirez-Campillo et al., 2013; Kibele et al., 2014; Granacher et al., 2015). In addition, the PJT was immediately performed after the warm-up program (Ramirez-Campillo et al., 2018c), participants used individualized box heights (i.e., 5–35 cm) during DJs (Ramirez-Campillo et al., 2018b), the order of drills was randomized during each week to provide variation to training and to avoid monotony (Ramirez-Campillo et al., 2018c), and the volume of training was increased progressively (Ramirez-Campillo et al., 2015c), with a high investigator to participant ratio of 1:5 realized during all PJT sessions (Ramirez-Campillo et al., 2016b), using an adequate rest between sets (Ramirez-Campillo et al., 2014a) and repetitions (Ramirez-Campillo et al., 2014b). Moreover, all PJT sessions were conducted in the specific training surfaces were players usually trained and compete (i.e., grass, land-dirt). In this sense, taking previous PJT research into consideration, mostly from soccer athletes, a highly specialized PJT program was applied in order to maximize adaptations. However, considering that the effects of PJT may vary according to sex, sport level, age, and years of experience (De Villarreal et al., 2009), current results may vary depending on the specific traits of the athletes.

From a practitioner's point of view, one PJT session per week in combination with soccer-specific training appears to be sufficient to induce performance improvements in female soccer players. A once per week PJT allows coaches and strength and conditioning professionals to save time that can be used for the performance of technical-tactical drills. In addition, this once per week PJT protocol may particularly allow amateur soccer players who cannot fully focus on their sport to complete a safe and effective PJT program. Whether more than two sessions per week may even induce greater improvements compared with one or two sessions per week needs to be elucidated in future studies. In addition, it is unclear whether our findings translate to young female players, professional female players, or male soccer players, or another type of sport, as the effects of PJT may vary according to such factors (De Villarreal et al., 2009; Saez-Saez De Villarreal et al., 2010; Saez De Villarreal et al., 2012; Asadi et al., 2017; Moran et al., 2017). Therefore, more research is needed in this area.

Some potential limitations should be acknowledge. Firstly, although several of the physical fitness measurements collected in the current study are significantly related to physiological and biomechanical parameters and competitive success (Arnason et al., 2004), a possible limitation of the present study was the absence of more physiological assessments to better understand the underlying mechanisms of PJT induced adaptations in female soccer players. Secondly, although PJT can improve physical fitness in female soccer players, to optimize training adaptations, this training strategy should be adequately applied in a more complex training plan that incorporates other explosive (e.g., sprints), endurance, technical, and tactical-oriented training methods. Finally, RPE-values were collected only in two training sessions, and mean values indicated potential differences between the groups. However, differences between the groups were expected, since we replaced a part of the habitual training strategy of participants with PJT. Specifically, the PJT drills replaced some low-intensity technical-tactical soccer drills. In this sense, it was expected that the PJT-1 and PJT2 groups achieved greater RPE, as these groups used PJT drills that stressed greater effort compared to low-intensity technical-tactical drills used by the control group. Moreover, it was also expected a relatively greater RPE from the PJT-1, since this group completed twice the volume of jumps per session as compared to the PJT-2 groups. However, when a statistical analysis was performed, the soccer training load (Table 1) did not achieves a significant difference (*p* = 0.2 to *p* = 0.5) between the groups.

In summary, findings from the present study demonstrated that a higher PJT frequency has no extra effect on female soccer players' physical fitness development during an 8 week PJT with moderate overall training volume. In other words, a different distribution (1 or 2 sessions) of a given weekly training volume (i.e., 70–140 jumps) does not produce statistically significant differences in training adaptations (CMJ, DJ20, MKV, 15-m sprint, CoDA, and Yo-YoIR1) in amateur female soccer players during an 8 week in-season PJT.

## Author contributions

RR-C, HC, and UG designed the work. RR-C: data acquisition. RR-C FG-P, AG-R, JY, PG, HC, and UG analysis and interpretation of data. RR-C FG-P, AG-R, JY, PG, HC, and UG: drafting the work. RR-C, FG-P, AG-R, JY, PG, HC, and UG: revising critically the work. RR-C, FG-P, AG-R, JY, PG, HC, and UG: final approval of the version to be published. RR-C, FG-P, AG-R, JY, PG, HC, and UG: agree to be accountable for all aspects of the work in ensuring that questions related to the accuracy or integrity of any part of the work were appropriately investigated and resolved.

### Conflict of interest statement

The authors declare that the research was conducted in the absence of any commercial or financial relationships that could be construed as a potential conflict of interest.
